# Preparation of a Highly Flame-Retardant Urea–Formaldehyde Resin and Flame Retardance Mechanism

**DOI:** 10.3390/polym16131761

**Published:** 2024-06-21

**Authors:** An Wei, Meifeng Ou, Shunxiang Wang, Yongjin Zou, Cuili Xiang, Fen Xu, Lixian Sun

**Affiliations:** 1 College of Materials Science and Engineering, Guilin University of Electronic Technology, Guilin 541004, China; wa123@mails.guet.edu.cn (A.W.); shxi3210@163.com (S.W.); sunlx@guet.edu.cn (L.S.); 2 Nanning Guidian Electronic Technology Research Institute Co., Ltd., Nanning 530000, China

**Keywords:** bio-based flame-retardants, phytic acid, chitosan, flame-retardant urea–formaldehyde resin, flame-retardant mechanism

## Abstract

Urea–formaldehyde (UF) resin is the most widely used adhesive resin. However, it is necessary to improve its flame-retardant performance to expand its applications. In this study, exploiting electrostatic interactions, anionic phytic acid and cationic chitosan were combined to form a bio-based intumescent flame-retardant, denoted phytic acid–chitosan polyelectrolyte (PCS). The molecular structure of the urea–formaldehyde resin was optimized by crosslinking with melamine and plasticizing with polyvinyl alcohol-124. Thus, by combining PCS with the urea–formaldehyde resin and with ammonium polyphosphate and ammonium chloride as composite curing agents, flame-retardant urea–formaldehyde resins (FRUFs) were prepared. Compared to traditional UF resin, FRUF showed excellent flame retardancy and not only reached the UL-94 V-0 level, but the limit of oxygen index was also as high as 36%. Compared to those of UF, the total heat release and peak heat release rate of FRUF decreased by 86.44% and 81.13%, respectively. The high flame retardancy of FRUF originates from the combination of oxygen and heat isolation by the dense carbon layer, quenching of phosphorus free radicals, and dilution of oxygen by a non-flammable gas. In addition, the mechanical properties of the FRUF remained good, even after modification. The findings of this study provide a reference for the flame-retardant application of FRUF for applications in multiple fields.

## 1. Introduction

As an important thermosetting resin, urea–formaldehyde (UF) resin is widely used in artificial board adhesives owing to its abundance, low cost, ease of use, good bonding performance, and colorless curing adhesive layer. It is the most widely used synthetic resin adhesive in the wood-processing industry [1,2,3,4]. In addition, UF can be applied in textile bonding and coatings [5]. However, with the increasing demand for fire safety, the inherent flammability of UF resin is a significant limitation, requiring the addition of flame retardants to improve its performance [6,7]. 

Halogen flame retardants have been widely used owing to their efficient flame retardancy and low impact on the mechanical properties of materials. However, these materials can produce harmful substances, such as toxic dioxins, during combustion, causing harm to the environment [8,9]. Therefore, there is demand for halogen-free flame retardants which have high flame-retardant efficiencies and environmental friendliness [10,11,12]. Current, intumescent flame retardants composed of phosphoric acid, carbon, and nitrogen gas sources are widely used owing to their advantages of low smoke toxicity, sustainability and high flame-retardant efficiency [13,14]. In addition, bio-based flame retardants have attracted the attention of researchers because of their environmentally friendly and biodegradable properties [15,16,17]. Biomass-based materials such as phytic acid (PA) [18,19], chitosan (CS) [20,21], lignin [22,23], cashew phenol [24], and eugenol [25] have been used to prepare bio-based flame retardants with good flame retardancy, which result from their unique structures. Notably, these bio-based materials can be modified to increase their contents of phosphorus, carbon, and nitrogen, and to obtain green and efficient bio-based intumescent flame-retardants [26,27,28].

PA is a phosphorus-rich type of biomass containing 12 negatively charged phosphate groups that provide strong chelating ability and can easily polymerize with positively charged cations or metal ions to form polyelectrolytes and metal salts [29]. In particular, because of its high content of phosphate groups, PA can be used as an acid source for intumescent flame-retardants, and it can promote the dehydration and carbonization of the matrix during the combustion process. Although flame-retardants such as cyclophosphazene can also endow materials with high flame retardancy [30], phytic acid has a higher phosphorus content and is more environmentally friendly. As a result, PA is widely used in flammable materials such as epoxy resins [31], polyurethane [32], and polylactic acid [33]. CS is another biomaterial that is rich in carbon and nitrogen and can act as a carbon and air source in an intumescent flame-retardant [34]; further, as a positively charged polymer, CS can combine with negatively charged PA to form a phytic acid–chitosan polyelectrolyte (PCS) rich in phosphorus and nitrogen [35,36]. As an additive, melamine is widely used for the modification of UF resins. It can block the hydrophilic groups of UF resin, effectively improving its water resistance. Simultaneously, it acts as a nitrogen source for flame-retardant systems. Polyvinyl alcohol-124 has good mechanical properties and can be used for toughening UF resin [37]. 

In this study, a bio-based flame-retardant, PCS, was synthesized via an ion crosslinking reaction using phytic acid and chitosan. Subsequently, the UF resin was optimized by modification with melamine and polyvinyl alcohol-124. Finally, the PCS was added to the optimized UF resin, and a flame-retardant UF resin (FRUF) was prepared using ammonium polyphosphate and ammonium chloride as composite curing agents. The flame-retardant performance of FRUF was studied and the flame-retardant mechanism was analyzed. In addition, the impact strength was studied. The flame-retardant urea–formaldehyde resin prepared in this study has high flame-retardant performance and can effectively achieve its flame-retardant applications in adhesives, coatings, and other fields.

## 2. Experimental Section

### 2.1. Materials

Phytic acid (PA, 70 wt.% in water), chitosan (CS, deacetylation degree ≥ 95%), formaldehyde solution (37 wt.% in water), melamine (AR), polyvinyl alcohol-124 (PVA-124; AR), ammonium polyphosphate (APP; AR), ammonium chloride (AR), and dioctyl phthalate (DOP; AR) were obtained from Aladdin Reagent Co. (Shanghai, China), and urea (AR) was obtained from Xilong Reagent (Shenzhen, China). Sodium hydroxide solution (20 wt.% in water) and acetic acid solution (20 wt.% in water) were both self-made in the laboratory.

### 2.2. Preparation

#### 2.2.1. Preparation of PCS

First, CS (5 g) was added to 2 wt.% acetic acid (400 mL) solution and mechanically stirred at 400 rpm for 3 h to obtain a clear CS solution. Then, while stirring at 800 rpm, 70 wt.% PA solution (5 mL) was added dropwise to the CS solution to obtain a PA-CS mixture. Then, the PA-CS mixed solution was mechanically stirred at 300 rpm for 3 h. Subsequently, the PA-CS mixed solution was filtered, and the filter residue was washed with deionized water until the pH of the washing solution was 4.0–5.0. Finally, the filter residue was freeze-dried for 24 h and ground into a powder to obtain the PCS. 

#### 2.2.2. Preparation of Modified UF Resin (MUF)

Firstly, 37 wt.% formaldehyde solution (100 g) was added to a three-necked flask, the pH of the solution was adjusted to 8.0–8.5 using sodium hydroxide solution, and the solution was mechanically stirred with heating to 90 °C. Then, the first batch of urea (37 g) was added at this temperature, followed by melamine (0.57 g) and polyvinyl alcohol-124 (0.86 g). Then, the temperature was kept constant and stirring was continued for 30 min. Subsequently, the pH was adjusted between 4.5 and 5.0 using an acetic acid solution, and the second batch of urea (12.3 g) was added. Then, the solution was stirred for 10 min until it became turbid. After the solution had become completely turbid, the pH was adjusted to 7.5–8.0 using a sodium hydroxide solution, and the third batch of urea (7.6 g) was added. The temperature was then reduced to 70 °C and stirred for 30 min. The obtained product was cooled to room temperature for storage to obtain a modified UF resin (MUF). The pure urea–formaldehyde resin without melamine or polyvinyl alcohol-124 modification is referred to as PUF.

#### 2.2.3. Preparation of Flame-Retardant Urea–formaldehyde Resin (FRUF)

Firstly, PCS was added to MUF in a mass ratio of 0 wt.%, 0.5 wt.%, and 1 wt.%. Subsequently, DOP and the co-curing agents ammonium chloride and ammonium polyphosphate were added to MUF in a mass ratio of DOP:NH_4_Cl:APP:MUF = 1:0.5:0.5:100. The mixture was stirred at 600 rpm for two hours to ensure even mixing of the components in MUF. The mixed solution was placed in the mold and cured at room temperature for 72 h to obtain flame-retardant urea–formaldehyde resin (FRUF). In addition, urea–formaldehyde resin (UF) without flame-retardant components was also prepared. The formulations used are listed in Table 1. 

### 2.3. Characterization

*Structural characterization:* A Fourier transform infrared (FTIR) spectroscopy test was performed using the TENSOR27 infrared spectrometer (BRUKER, Saarbrücken, Germany) to analyze the structure of PCS. The microstructure of the resin residual carbon layer was observed through a Quanta 450 FEG field emission scanning electron microscope (FEI, Hillsboro, OR, USA). The degree of graphitization of the resin residue was analyzed using a HORIBA LabRAM HR Evolution Raman spectrometer (HORIBA Scientific, Paris, France) at a laser excitation wavelength of 532 nm. The gaseous decomposition products of the sample were analyzed using a Bruker TG-FTIR (BRUKER, Saarbrücken, Germany) infrared thermogravimetric analysis (TG-IR).

*Thermal analysis:* The curing behavior of the material was analyzed by a DSC-250 (TA Instruments, New Castle, DE, USA) differential scanning calorimeter (DSC) under nitrogen atmosphere. The temperature range was 40–180 °C, and the heating rate was 10 °C/min. Thermo Fisher Is-50 thermogravimetric analyzer (Thermo Fisher Scientific, Waltham, MA, USA) was used for thermogravimetric analysis (TG). The temperature range was 50 to 800 °C, and the heating rate was 20 °C/min; the nitrogen flow rate was 100 mL/min.

*Flame retardance:* A UL-94 vertical combustion test was performed using a CZF-3 vertical combustion test instrument (Jiangning Analytical Instrument Co., Ltd., Nanjing, China). The sample size was 130 mm × 13 mm × 3 mm. A HC-2 oxygen index meter (Jiangning Analytical Instrument Co., Ltd., Nanjing, China) was used for ultimate oxygen index testing. The sample size was 130 mm × 6.5 mm × 3 mm. The cone calorimetry test was conducted using a TTech-GBT161172 cone calorimeter (Testech Technology, Suzhou, China). The sample size was 100 mm × 100 mm × 3 mm.

*Mechanical performance:* The impact strength of the flame-retardant urea–formaldehyde resin was tested using a simply supported beam impact tester (Shanghai Hesheng Instrument Technology, Shanghai, China).

## 3. Results and Discussion 

### 3.1. Structural Characterization of PCS

**Figure 1:**
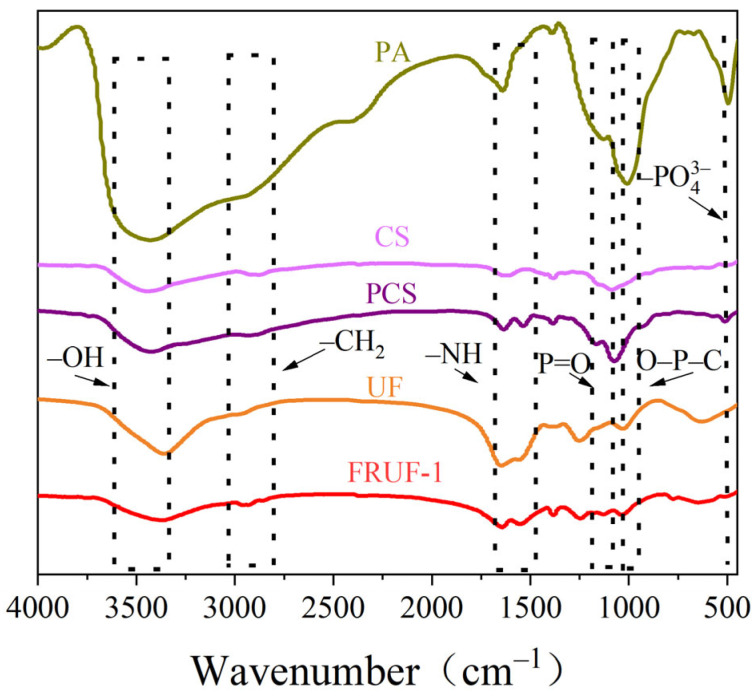
FTIR spectra of PA, CS, PCS, UF, and FRUF-1.

Figure 1 shows the FTIR spectra of PA, CS, and PCS. For CS, bands corresponding to the stretching vibrations of -OH and -CH_2_ were observed at 3450 and 2876 cm^−1^, respectively, and a bending vibration belonging to -NH was observed at 1650 cm^−1^ [35]. Notably, the intensity of the -CH_2_ band at 2876 cm^−1^ in CS was reduced in the spectrum of PCS, suggesting that hydrogen bonding was formed between the methylene-linked hydroxyl group in CS and the phosphate group in PA [38]. For PA and PCS, the characteristic bands at 1190 and 1059 cm^−1^ correspond to the tensile vibrations of P=O and O-P-C. Compared with that of CS, PCS shows a new absorption peak band at 501 cm^−1^; this may come from -PO_4_^3−^ in PA [39]. According to the analysis, it can be inferred that PA and CS reacted and PCS was successfully synthesized. In addition, compared with UF, new peaks such as P-O and P=O from PCS were observed in FRUF-1, indicating that PCS was successfully added to the resin.

### 3.2. Thermal Properties of Resin

**Figure 2:**
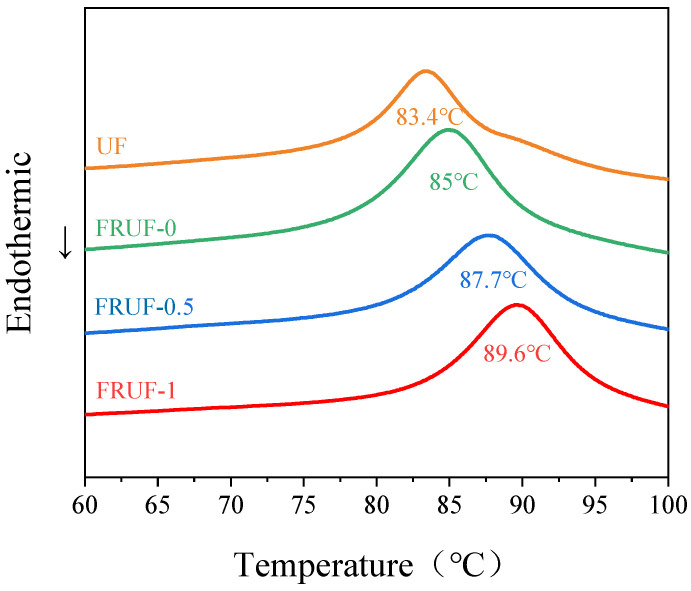
DSC curves of different resin samples.

As shown in Figure 2, the overall trend of the DSC exothermic peaks for different resins was the same, with a clear exothermic peak appearing, indicating that the optimization of the structure, addition of composite curing agents, and addition of PCS did not change the overall curing behavior of the resin. Compared with that of UF, the peak temperature of the exothermic peak of FRUF-0 only increased by 1.6 °C. Compared with that of FRUF-0, the peak temperature of FRUF-0.5 increased by 2.7 °C, whereas that of FRUF-1 increased by 4.6 °C, indicating that the addition of PCS increases the temperature for resin curing. This is because PCS, as a large molecule, hinders the movement of the resin macromolecular chains, leading to a decrease in reactivity. 

**Figure 3:**
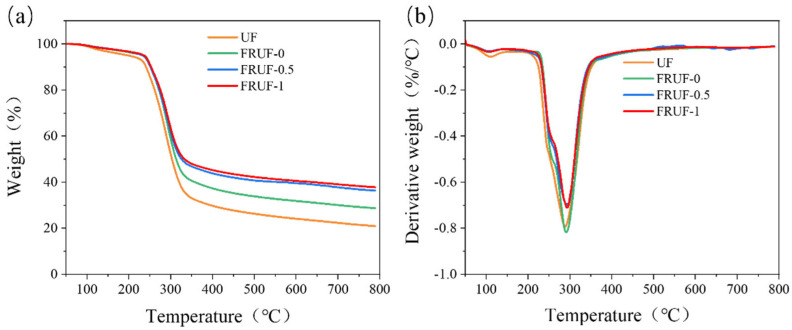
TG (**a**) and DTG (**b**) curves for different resin samples.

The TG results for the different resins are presented in Figure 3 and Table 2. As shown, the structure optimization, use of a composite curing agent, and addition of PCS did not change the thermal decomposition process of the UF resin. Further, after structure optimization and curing with the composite curing agent, the temperature of the resin at a decomposition mass of 5% increased significantly from 197.3 to 237.6 °C, mainly because of the high decomposition temperature of ammonium polyphosphate contained in the composite curing agent. However, after the addition of PCS, the decomposition temperature of the resin decreased slightly, but with the further increase in PCS content, the decomposition temperature began to increase. Overall, the optimized structure and composite curing agent had little effect on the temperature at which the resin reached its maximum decomposition rate, whereas the addition of PCS resulted in an increase in the temperature at which the resin reached its maximum decomposition rate. Notably, structure optimization and the addition of composite curing agents and PCS significantly improved the thermal stability of the resin. In addition, the residual carbon content of FRUF-0 increased by 7.8 wt.% compared to that of UF at 800 °C, and the carbon residue of FRUF-1 with 1 wt.% PCS was as high as 37.8%, almost twice that of UF. The increase in the residual carbon content is attributed to the composite curing agent and the phosphate groups in the PCS, which promote matrix dehydration into carbon during the decomposition process, thereby protecting the matrix.

### 3.3. Flame Retardancy

**Figure 4:**
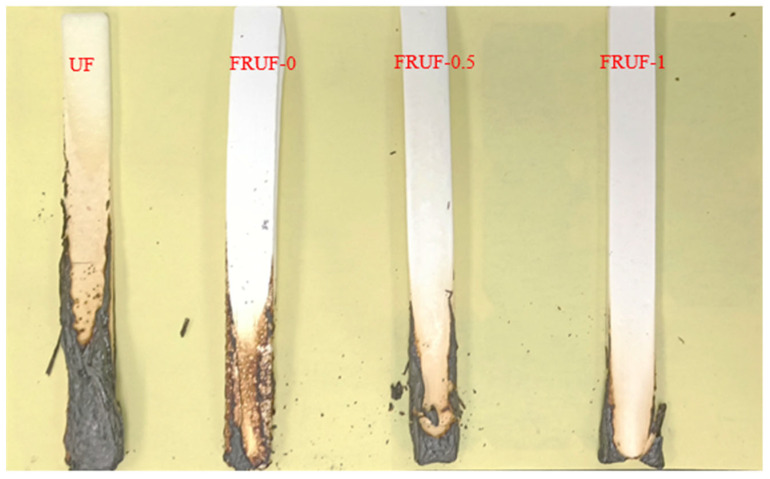
Photo after the vertical burning experiment.

To demonstrate the flame-retardant performance of the material, UL-94 experiments and LOI tests were conducted. The UL-94 and LOI experimental results for the flame-retardant urea–formaldehyde resin are shown in Figure 4 and Table 3, respectively. The UF only reached the V-1 level in the UL-94 experiment with an LOI of 29.5%. FRUF-0 successfully passed the UL-94 V-0 grade test, and the LOI increased to 32.1%. After the addition of PCS, the ultimate oxygen index of the resin increased further, and the addition of 1 wt.% resulted in an ultimate oxygen index of 36%. With the optimization of the structure, use of composite curing agents, and an increase in the PCS content, the area of resin burning decreased. In the vertical combustion experiments, the UF burned to the middle and collapsed slightly in the lower half, whereas FRUF-1 burned only slightly at the bottom, as shown in Figure 4. 

**Figure 5:**
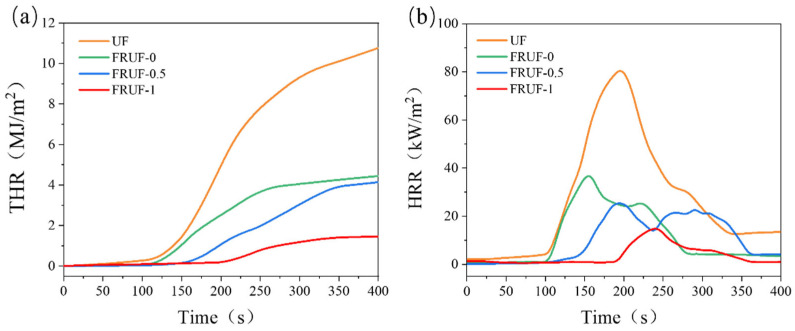
THR (**a**) and HRR (**b**) plots of different samples.

To simulate the combustion of the samples, cone calorimetry tests were conducted. The results and parameters are shown in Figure 5 and Table 4, respectively. Although the time to ignition (TTI) only increased by 1 s, the total heat release (THR) of FRUF-0 decreased by 58.76% compared with that of UF, and the peak heat release rate (PHRR) also decreased by 54.19%. This indicates that the optimization of the UF resin structure and the use of composite curing agents improved the flame-retardant performance of the UR resin. After adding PCS, the TTI of the FRUF resin was significantly increased, whereas the THR and PHRR decreased. On increasing the amounts of PCS from 0 to 0.5 wt.%, the TTI increased from 135 to 167 s. At 1 wt.% PCS, the TTI of FRUF-1 was 220 s, but the THR and PHRR decreased relative to UF by 86.44% and 81.13%, respectively. These results indicate that PCS not only reduces the heat released by the UF resin during combustion but also significantly prolongs the time required for its ignition. The fire growth index (FGI) reflects the fire hazard level of materials, and a lower FGI indicates a lower fire hazard level [40]. Compared with UF, the FGI of FRUF-1 decreased by 85.37%, indicating that the fire hazard of the FRUF resin was significantly lower than that of unmodified UF.

**Figure 6:**
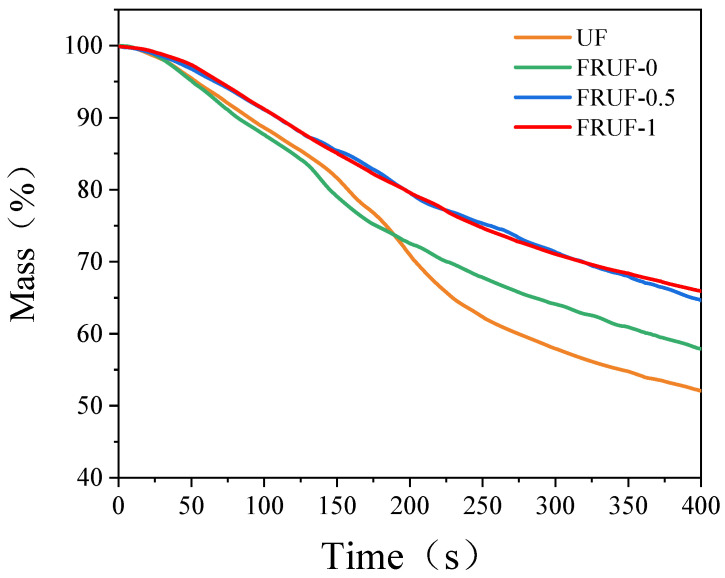
Mass loss during cone calorimetry testing.

The mass losses during cone calorimetry tests are shown in Figure 6. As shown, the FRUF samples have increased residues compared to UF. That is, adding PCS increased the post-combustion residue. The results are consistent with TG testing.

### 3.4. Mechanism of Flame Retardancy

#### 3.4.1. Residual Carbon Analysis

**Figure 7:**
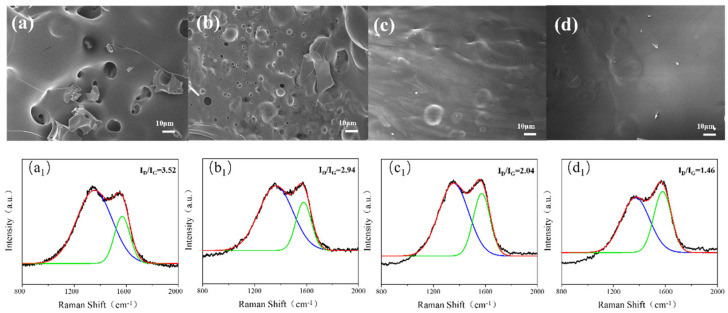
SEM images and Raman spectrum of residual carbon of various resin samples after combustion: UF (**a**,**a_1_**), FRUF-0 (**b**,**b_1_**), FRUF-0.5 (**c**,**c_1_**), and FRUF-1 (**d**,**d_1_**).

Next, SEM and Raman tests were performed on the carbonaceous residue after the complete combustion of the UF resin. Figure 7a reveals the presence of numerous large pores within the carbon residue, attributable to gaseous emissions during combustion, as well as many large fissures. Subsequent structural optimization and incorporation of the composite curing agent resulted in a more compact carbon residue from FRUF-0, which showed a reduced pore size, probably as a result of the phosphate moiety of ammonium polyphosphate (Figure 7b). The addition of PCS resulted in an absence of pores in the carbon residue of FRUF-0.5, although some surface wrinkling persisted (Figure 7c). Increasing the PCS content to 1 wt.% yielded a smoother carbon residue in FRUF-1 (Figure 7d). These observations indicate the formation of a dense carbon layer on the condensed phase of the FRUF resin throughout combustion, effectively insulating against oxygen and heat, thereby enhancing flame retardancy [41]. 

The Raman spectra confirm the increase in the density of the carbon layer within the resin residue. The *I*_D_/*I*_G_ peak ratio in Raman spectra reveals the degree of graphitization, for which a lower ratio suggests a higher degree of graphitization. As shown in Figure 7a_1_, the *I*_D_/*I*_G_ ratio was 3.52, indicating minimal graphitization. Conversely, the *I*_D_/*I*_G_ ratio of FRUF-1 in Figure 7d_1_ is 1.46, indicating a substantially higher degree of graphitization compared to UF. The results of the Raman test correspond to those of the SEM test.

#### 3.4.2. Gas Phase Product Analysis

**Figure 8:**
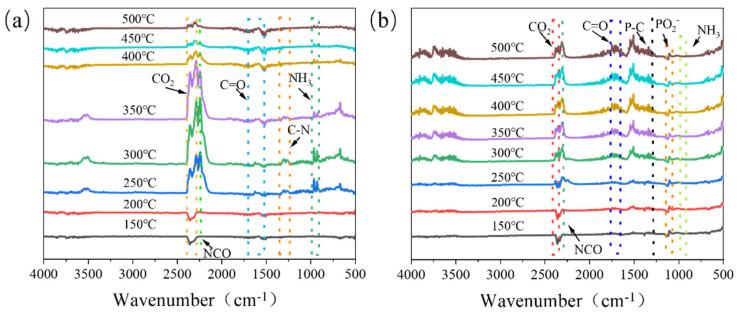
Infrared spectra of UF (**a**) and FRUF-1 (**b**) decomposition products at different temperatures.

The thermal decomposition products of flame-retardant resins can be analyzed using TG-IR. Figure 8a indicates that the primary gaseous products include CO_2_ (2360 cm^−1^), NCO (2265 cm^−1^) [42], carbonyl compounds (1627 cm^−1^), and NH_3_ (964 cm^−1^). Figure 8b shows that, in addition to the pyrolysis products of UF, those of FRUF-1, as revealed by new spectral peaks, were phosphorus-based substances, such as P-C (1265 cm^−1^) and PO_2_^−^ (1140 cm^−1^). A comparative analysis of the spectral peaks of UF and FRUF-1 revealed a marked reduction in the intensity of the CO_2_ and NH_3_ signals for FRUF-1 between 200 and 400 °C, suggesting reduced gaseous emissions during combustion. This may be due to the phosphate groups present in PCS and APP, which likely facilitated carbon layer formation during combustion, thereby protecting the underlying substrate and mitigating pyrolysis. 

**Figure 9:**
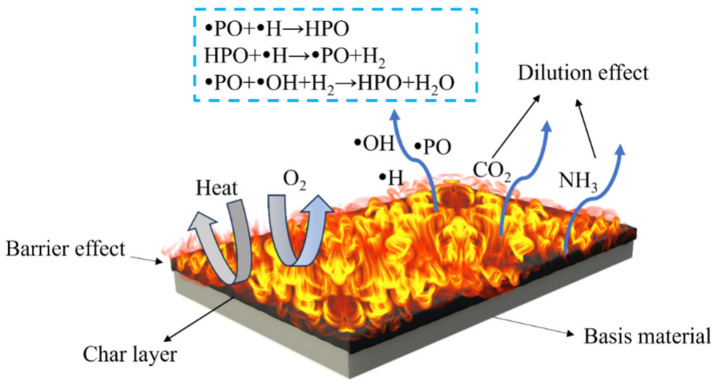
Schematic of the potential flame-retardant mechanism of flame-retardant urea–formaldehyde resin.

The aforementioned analyses suggest that the flame-retardant mechanism of the FRUF resin is as follows: a dense carbon layer forms on the condensed phase, isolating oxygen and combustion-generated heat. In the gas phase, non-combustible gases dilute oxygen, whereas phosphorus sequesters atmospheric free radicals, decelerating combustion chain reactions and reducing the extent of combustion [43]. This mechanism typifies an intumescent flame-retardant process, as depicted in Figure 9. 

### 3.5. Mechanical Properties

**Figure 10:**
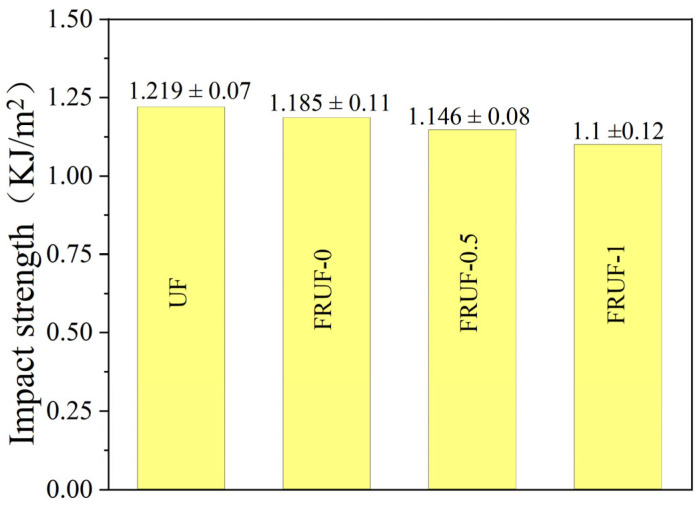
Impact strength assessment of different resin samples.

Next, the impact resistance of the FRUF resin was evaluated, and the results are shown in Figure 10. The UF resin exhibited an impact strength of 1.219 kJ/m^2^, whereas the FRUF-0 variant showed a slight reduction to 1.185 kJ/m^2^. The incorporation of PCS led to a further decrease in the impact strength and was directly proportional to the PCS concentration. Nonetheless, the FRUF-1 variant maintained an impact strength of 1.1 kJ/m^2^, equivalent to 90.2% of the impact resistance of the original UF resin. This suggests that the mechanical integrity of the FRUF resin is largely preserved, making it suitable for real-world applications.

## 4. Conclusions

In this study, we synthesized a bio-based flame retardant, PCS, utilizing high-phosphorus phytic acid, which is known for its potent chelating properties, and positively charged chitosan derived from biomass. A series of flame-retardant UF resins exhibiting enhanced flame resistance was developed by modifying UF resins with PCS, melamine, and polyvinyl alcohol-124, and employing ammonium polyphosphate and ammonium chloride as composite curing agents. The modified UF resins outperformed the unaltered UF resin, increasing from a V-1 to a V-0 rating and achieving an LOI of 36%. Concurrently, there was a significant reduction in HRR and PHRR of 86.44% and 81.13%, respectively, and the FGI decreased by 85.37%. A thorough investigation into the flame-retardant mechanism of UF resin indicated that its superior flame resistance is attributed to an intumescent flame-retardant mechanism. Moreover, the impact resistance of the flame-retardant UF resin was maintained at 90.2% of the UF resin, indicating the preservation of its mechanical properties. However, the impact strength of the resin is not high and needs to be further improved. At the same time, the influence of PCS on other mechanical properties of the resin also needs to be perfected in subsequent studies. This study offers innovative perspectives for incorporating bio-based flame retardants into UF resins. The prepared flame-retardant urea–formaldehyde resin has high flame-retardant performance and can effectively achieve its flame-retardant applications in adhesives, coatings, and other fields.

## Figures and Tables

**Table 1:** Formulations of flame-retardant urea–formaldehyde resin.

Sample	PUF (g)	MUF (g)	PCS (g)	DOP (g)	NH_4_Cl (g)	APP (g)
UF	100	-	-	1	1	-
FRUF-0	-	100	-	1	0.5	0.5
FRUF-0.5	-	100	0.5	1	0.5	0.5
FRUF-1	-	100	1	1	0.5	0.5

**Table 2:** Test results of TG.

Sample	*T*_d5%_ (°C)	*T*_peak_ (°C)	*R*_800_ (wt.%)
UF	197.3	287.4	20.9
FRUF-0	237.6	288.5	28.7
FRUF-0.5	233.6	298.9	36.3
FRUF-1	237.3	294.1	37.8

**Table 3:** UL-94 vertical burning and LOI data for the samples.

Sample	LOI (%)	*t*_1_	*t*_2_	Dropping	UL-94 Rating
UF	29.5	11.9 s	14.4 s	No	V-1
FRUF-0	32.1	4.6 s	7.1 s	No	V-0
FRUF-0.5	34.4	1.9 s	2.5 s	No	V-0
FRUF-1	36	0.1 s	0.3 s	No	V-0

**Table 4:** Cone calorimeter data (heat flux: 25 kW/m^2^).

Sample	TTI (s)	PHRR (kW/m^2^)	THR (MJ/m^2^)	TSP (m^2^)	AV-EHC (MJ/kg)	FGI (kW/m^2^/s)
UF	134 ± 9	81.01 ± 5.47	10.77 ± 0.14	278.45 ± 12.15	10.11 ± 1.31	0.41 ± 0.03
FRUF-0	135 ± 7	37.19 ± 3.51	4.44 ± 0.11	254.14 ± 15.18	6.07 ± 0.15	0.24 ± 0.02
FRUF-0.5	167 ± 8	25.42 ± 2.77	4.14 ± 0.15	126.74 ± 13.14	5.43 ± 0.12	0.13 ± 0.01
FRUF-1	220 ± 10	15.29 ± 1.17	1.46 ± 0.12	115.93 ± 7.19	2.12 ± 0.06	0.06 ± 0.01

## Data Availability

The original contributions presented in the study are included in the article, further inquiries can be directed to the corresponding author.
